# Population Genetic Data for 23 STR Loci of the Black Caribbean Ethnic Group in Honduras

**DOI:** 10.3390/genes17050496

**Published:** 2026-04-22

**Authors:** Antonieta Zuniga, Yolly Molina, Karen Amaya, Zintia Moya, Patricia Soriano, Digna Pineda, Yessica Pinto, Oscar Garcia, Isaac Zablah

**Affiliations:** 1 Dirección de Medicina Forense, Ministerio Público, Calle la Salud, Tegucigalpa 11101, Honduras; antonietazuniga2311@yahoo.com (A.Z.); yolly.molina@unah.edu.hn (Y.M.); kamayapach@gmail.com (K.A.); zintiamoya@gmail.com (Z.M.); paty.soriano@yahoo.com (P.S.); dignapineda101969@yahoo.com (D.P.); yepico811@yahoo.com (Y.P.); 2 Center for Biomedical Imaging Diagnostics Research and Rehabilitation, National Autonomous University of Honduras, Blvd. Suyapa, Tegucigalpa 11101, Honduras; 3 Basque Country Forensic Genetics Laboratory, Larrauri Mendotxe 18, 48950 Erandia, Bizkaia, Spain; ogarcia@seg.euskadi.eus; 4 Faculty of Medical Sciences, National Autonomous University of Honduras, Calle la Salud, Tegucigalpa 11101, Honduras

**Keywords:** short tandem repeats (STRs), population genetics, Black Caribbean, Honduras, forensic genetics, PowerPlex Fusion 6C, Afro-descendant, allele frequencies

## Abstract

Background/Objectives: The Black Caribbean population of Honduras, also referred to locally as Negro Inglés, constitutes one of the country’s nine recognized indigenous and Afro-descendant peoples. Predominantly settled in the Bay Islands and sections of the Caribbean coast, this community traces its ancestry predominantly to West Africa and has remained culturally and linguistically distinct for more than three centuries. Despite its demographic and historical relevance, no population-specific short tandem repeat (STR) database has been established for this group. Methods: Allele frequencies for 23 autosomal STR loci were characterized in 100 unrelated Black Caribbean individuals from the department of Islas de la Bahía. DNA was extracted from blood on FTA cards and amplified with the PowerPlex Fusion 6C System (Promega Corporation). Statistical parameters were computed using Genepop v4.2, Arlequin v3.5 and GDA v1.0. Results: A total of 241 distinct alleles were detected across all 23 loci (mean 10.48 ± 3.85 alleles/locus). Expected heterozygosity ranged from 0.6541 (D13S317) to 0.9350 (SE33), with a mean of 0.8150 ± 0.0664—values consistent with a population of predominantly West African origin. No locus exhibited a significant departure from Hardy–Weinberg equilibrium after Bonferroni correction (α = 0.0022). The combined power of discrimination exceeded 99.9999% and the combined chance of exclusion surpassed 99.9999%. Conclusions: This first genetic characterization of the Honduran Black Caribbean population delivers an essential, population-specific reference dataset for forensic casework, paternity testing, and population genetics research. The data also deepen the understanding of Afro-descendant genetic diversity in Central America and constitute a critical step towards equitable forensic genetic services for all Honduran ethnic communities.

## 1. Introduction

The establishment of high-quality population-specific allele frequency databases is a prerequisite for statistically sound and legally defensible forensic DNA interpretation. Estimates of random match probability (RMP), likelihood ratios (LRs), and paternity indices (PI) are derived from allele frequencies; accordingly, the choice of reference database can materially affect the weight assigned to genetic evidence, particularly in structured or admixed populations [1,2,3,4]. This issue is particularly relevant in multi-ethnic nations such as Honduras, where nine legally recognized indigenous and Afro-descendant peoples co-exist alongside a large mestizo majority, yet forensic databases remain incomplete for most of these groups.

Honduras formally recognizes nine indigenous and Afro-descendant peoples: the Lenca, Maya Chortí, Garifuna, Miskito, Tawahka, Pech, Tolupán, Negro Inglés (Black Caribbean), and the Nahua [5]. Of these, published autosomal STR databases exist only for the Lenca [6] and, most recently, the Tawahka [7]. The remaining groups, including the Black Caribbean population, still lack population-specific forensic reference data. In practice, this gap may require forensic laboratories to rely on the Honduran mestizo dataset or on broader Hispanic reference data associated with CODIS-based forensic resources [8,9], neither of which can be assumed to accurately represent the allele-frequency distribution of all Honduran minority populations.

**Figure 1:**
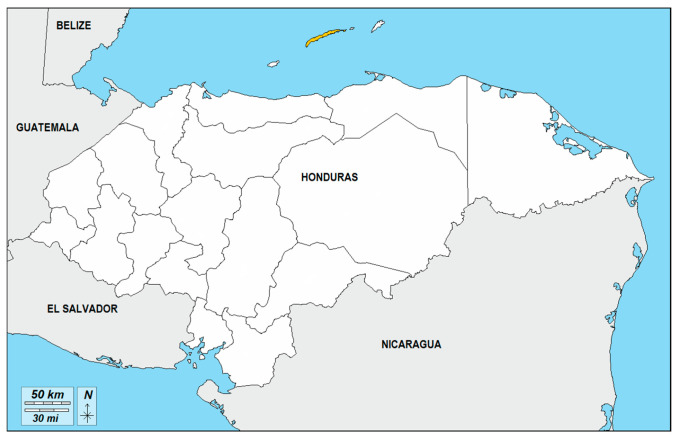
In yellow the main geographic distribution area of Black Caribbean at Roatan Island. Map modified from D-maps [12,13].

The Black Caribbean community in Honduras, widely known locally as Negro Inglés (Black English or English-speaking black) and rarely as Isleño (islander), is primarily settled in the Bay Islands department (Islas de la Bahía—Roatán, Utila, and Guanaja) and along portions of the northern Caribbean coast, see Figure 1. This population traces its origins predominantly to West and Central Africa via the British colonial slave trade during the seventeenth and eighteenth centuries [10]. Unlike the Garifuna, who emerged from the intermingling of Amerindian Arawak/Carib peoples and escaped Africans in Saint Vincent before forcible deportation to Central America, the Black Caribbean of Honduras descends more directly from enslaved Africans and shares a historical and cultural affiliation with English-speaking Afro-Caribbean societies of Jamaica, the Cayman Islands, and the Eastern Caribbean [11]. This distinct ethnohistorical trajectory provides a clear rationale for generating a dedicated allele-frequency database rather than extrapolating from mestizo, indigenous or Garifuna reference populations.

Short tandem repeat (STR) markers have remained the global standard for human forensic identification for more than three decades, owing to their high polymorphism, technical robustness, and the existence of internationally standardized panels that facilitate inter-laboratory comparison [14,15]. The PowerPlex Fusion 6C System (Promega Corporation, Madison, WI, USA) simultaneously co-amplifies 23 autosomal STR loci encompassing all 20 CODIS core loci, all 16 European Standard Set (ESS) loci, and the SE33 marker [16,17]. This validated multiplex is directly compatible with contemporary forensic casework and databasing workflows.

The present study pursues four primary objectives: (1) to establish the first population-specific autosomal STR allele frequency database for the Honduran Black Caribbean community; (2) to compute forensic statistical parameters—including PD, CE, and PIC—relevant to casework and paternity testing; (3) to evaluate Hardy–Weinberg equilibrium at all 23 loci; and (4) to contextualize the observed genetic diversity within the broader Central American and Afro-descendant genetic landscape. By characterizing a currently undocumented Afro-descendant population in Honduras, this study provides a population-specific reference resource that extends the analytical options available to local forensic laboratories, whilst acknowledging that its appropriate forensic application depends critically on the casework context and the composition of the relevant suspect pool [3,18,19].

## 2. Materials and Methods

### 2.1. Sample Collection

Blood samples were obtained from 100 unrelated, self-identified Black Caribbean individuals residing in the Bay Islands department of Honduras. Participant eligibility required self-reported Black Caribbean ancestry spanning at least three generational tiers, with no acknowledged first- or second-degree familial relationships with other enrolled participants. Demographic representation was sought across the principal islands of Roatán, Utila, and Guanaja. The sex distribution of the sample reflects voluntary participation rates in each community and the broader demographic composition of the population.

To confirm sample independence, three complementary verification strategies were employed: (1) structured interviews to elicit information on first- and second-degree kinship among participants; (2) confirmation that no two individuals carried identical 23-locus STR profiles; and (3) pairwise identity-by-state (IBS) values were calculated across the 23 autosomal STR loci to screen for potential cryptic first-degree relatives, as IBS/allele-sharing metrics have been shown to distinguish first-degree relatives from unrelated individuals in STR-based forensic datasets; all observed pairwise IBS values were consistent with the distribution expected for unrelated individuals from the same population [20].

### 2.2. DNA Extraction

Venous blood was collected by trained phlebotomists following standard venipuncture protocols [21] and immediately absorbed onto Indicating FTA Cards (Qiagen, Germantown, MD, USA). FTA technology facilitates room-temperature preservation of genomic DNA by lysing cells on contact, denaturing proteins, and protecting DNA from oxidative damage and nuclease activity [22,23]. Cards were stored at ambient temperature in dry, sealed packaging until amplification.

### 2.3. PCR Amplification

PCR amplification was performed directly on FTA card punches using the PowerPlex Fusion 6C System (Promega Corporation, Madison, WI, USA) in strict accordance with the manufacturer’s protocols [24]. This system simultaneously amplifies 27 loci: 23 autosomal STRs (CSF1PO, D1S1656, D2S441, D2S1338, D3S1358, D5S818, D7S820, D8S1179, D10S1248, D12S391, D13S317, D16S539, D18S51, D19S433, D21S11, D22S1045, FGA, Penta D, Penta E, SE33, TH01, TPOX, and vWA), three Y-chromosome STR loci (DYS391, DYS570, and DYS576), and the amelogenin gender marker.

### 2.4. Fragment Analysis and Genotyping

Amplified products were resolved by capillary electrophoresis on an Applied Biosystems 3500 Genetic Analyzer (Thermo Fisher Scientific, Waltham, MA, USA) and genotyped using GeneMapper v1.4 software. Allele designation followed guidelines issued by the DNA Commission of the International Society for Forensic Genetics (ISFG), using manufacturer-supplied allelic ladders as calibration standards and following the National Institute of Standards and Technology (NIST) database STRBase [25,26,27].

### 2.5. Statistical Analysis

Allele frequency estimation and population genetic statistics were computed in Genepop v4.2 [28] and Arlequin v3.5 [29]. Per-locus forensic parameters, power of discrimination (PD) and chance of exclusion (CE) were calculated following Huston [30]; polymorphic information content (PIC) was derived according to Botstein et al. [31]. Departures from Hardy–Weinberg equilibrium (HWE) [32] were assessed by exact tests implemented in the GDA v1.0 software [33]. To control family-wise error inflation attributable to 23 simultaneous tests, Bonferroni correction was applied, yielding a per-locus significance threshold of α = 0.0022 (0.05/23) [34]. Population differentiation was estimated using pairwise FST values calculated via the Weir and Cockerham [35] method in Arlequin, enabling a preliminary assessment of the genetic distance between the Black Caribbean population and other Honduran reference groups.

### 2.6. Principal Components Analysis

To provide a reviewer-oriented comparative framework without over merging heterogeneous datasets, we constructed a two-panel principal coordinates analysis (PCoA) based on pairwise FST distances. Panel A was reconstructed from harmonized published allele-frequency tables for the present Black Caribbean sample and five Afro-Atlantic reference populations: Akan (Ghana), African Americans, Bahamians, Jamaicans, and Trinidadians. To maximize inter-study comparability, this ordination was restricted to 11 shared autosomal STR loci: TH01, D3S1358, vWA, TPOX, D8S1179, D18S51, D16S539, CSF1PO, D13S317, D5S818, and D7S820. Panel B was constructed separately from published pairwise FST values among the Honduran Lenca, Tawahka, and Garifuna populations, all characterized with comparable 23-locus datasets. This two-panel design allowed integration of the broader Afro-Atlantic context with the Honduran reference context while preserving methodological comparability within each analytical block [6,7,19,36].

### 2.7. Quality Control

Laboratory procedures were conducted in accordance with the standards of GITAD (Ibero-American Working Group on DNA Analysis) [37]. Each amplification batch included at least one positive and one negative control. Allele calls were validated against the internal lane size standard and the allelic ladder to ensure accurate sizing. Stutter thresholds and peak height ratios were monitored throughout. Laboratory performance was independently verified through participation in external proficiency testing schemes, collectively ensuring the accuracy and reproducibility of the dataset.

Each amplification batch incorporated positive controls, namely the 9947A female and 2800M male DNA standards, as well as negative controls consisting of reagent blanks. All genetic profiles were independently reviewed and verified by a second analyst to ensure analytical reliability. Interpretation thresholds followed the manufacturer’s recommendations, including a detection threshold of 175 RFU for heterozygous peaks, locus-specific stutter filters, and an allele-sizing precision of ±0.5 bp. Whenever present, microvariant alleles (e.g., x.1, x.2, and x.3) were designated in accordance with ISFG nomenclature.

### 2.8. Ethics Statement

The study’s protocol was approved by the Biomedical Research Ethics Committee (CEIB) of the Scientific Research Unit (UIC), Faculty of Medical Sciences, National Autonomous University of Honduras (UNAH) (IRB-00003070), and was conducted in full compliance with the Declaration of Helsinki. All participants provided written informed consent prior to enrolment. Community leaders of each island settlement were consulted throughout the study design and implementation phases to ensure cultural appropriateness and collective acceptance. The study further adhered to the Nagoya Protocol on Access and Benefit-Sharing, ratified by Honduras in 2015, through prior informed consent, mutually agreed benefit-sharing terms, and transparent communication with community representatives.

## 3. Results

### 3.1. Dataset Overview

The dataset comprises allele frequency data for 23 autosomal STR loci derived from 100 unrelated Black Caribbean individuals. For each locus, observed allele designations and their frequencies are reported in Supplementary File S1 alongside sample size (N = 100), observed heterozygosity (Ho), expected heterozygosity under Hardy–Weinberg expectations (He), PD, CE, PIC, minimum allele frequency (MAF), and the HWE exact-test *p*-value.

### 3.2. Allelic Richness

Across the 23 STR loci, 241 distinct alleles were detected. Allelic richness per locus ranged from 6 alleles at D10S1248 and TH01 to 24 alleles at the hypervariable SE33 locus, with a mean of 10.48 ± 3.85 alleles per locus. The pentanucleotide marker Penta E harboured 15 alleles; FGA and D19S433 each presented 14 and 13 alleles, respectively. The profile of allelic diversity is notably elevated relative to previously characterized Honduran indigenous populations, a pattern consistent with the well-documented higher STR polymorphism typical of populations of predominantly West African ancestry [38].

### 3.3. Statistical Parameters

Expected heterozygosity spanned a broad range: 0.6541 (D13S317) to 0.9350 (SE33). Eight loci exhibited He > 0.85—SE33 (0.9350), Penta E (0.9042), D2S1338 (0.8858), Penta D (0.8843), D19S433 (0.8709), FGA (0.8673), D18S51 (0.8732), and D21S11 (0.8561)—underscoring the outstanding individual discrimination capacity of these markers in this population. A further 13 loci displayed moderate-to-high diversity (He 0.70–0.85), and only D13S317 (He = 0.6541) and D5S818 (He = 0.6946) showed values below 0.70. The mean observed heterozygosity (Ho = 0.8065 ± 0.0637) closely matched the mean expected value (He = 0.8150 ± 0.0664), indicating no systematic heterozygote deficit or excess at the panel level. Table 1 presents the full set of per-locus statistics. Key forensic indicators are summarized below.

Power of discrimination ranged from 0.8310 (D13S317) to 0.9806 (SE33) at the individual locus level, and the combined power of discrimination across all 23 loci, calculated under the product rule with theta correction (θ = 0.01) [3]; exceeded 99.9999%, corresponding to a combined random match probability (RMP) below 1 in 10^14^. The combined chance of exclusion surpassed 99.9999%, confirming the panel’s exceptional suitability for paternity testing. PIC values ranged from 0.5999 (D13S317) to 0.9261 (SE33), with 22 of 23 loci exceeding PIC = 0.60, the conventional threshold for a marker to be considered highly informative.

### 3.4. Hardy–Weinberg Equilibrium

Exact tests for departures from the Hardy–Weinberg equilibrium were conducted at all 23 loci. Prior to multiple-testing correction, a single locus—D12S391—returned a nominally significant *p*-value (*p* = 0.0320). After application of the Bonferroni-corrected significance threshold (α = 0.0022), this deviation no longer met the criterion for statistical significance. Accordingly, no locus exhibited a significant HWE departure after correction, consistent with a sample of unrelated individuals drawn from a population at genetic equilibrium and confirming the suitability of the dataset for generating unbiased forensic statistics.

## 4. Discussion

### 4.1. Allele Diversity and West African Genetic Signatures

The allelic richness and heterozygosity observed in the Black Caribbean population of Honduras are markedly elevated relative to indigenous Honduran groups and are consistent with the well-established principle that sub-Saharan African populations harbour the highest within-population genetic diversity of any human continental group [38]. With a mean He of 0.8150 ± 0.0664 and 10.48 alleles per locus on average, the Black Caribbean population substantially surpasses the Tawahka (He = 0.7385; 8.61 alleles/locus) [7] and the Lenca (He = 0.7425; 8.91 alleles/locus) [6], and also compares favourably with the admixed Guatemalan Ladino population (He = 0.7656; 9.45 alleles/locus) [16], whose diversity is partly attributable to European admixture. This elevated diversity is consistent with the demographic history of the Black Caribbean community, whose founding population derived from continental West Africa, a region characterized by exceptionally large effective population sizes and deeply divergent allele lineages at STR markers [38,39].

The locus-specific patterns observed in this dataset are broadly compatible with the demographic history of an African-descendant population, particularly when interpreted in light of the overall levels of heterozygosity, allelic richness, and the multilocus profile. However, caution is warranted when assigning ancestry-related meaning to individual forensic STR loci, because autosomal identification STRs are primarily optimized for individualization and kinship testing rather than fine-scale biogeographic ancestry inference. Although TH01 has been discussed in the ancestry literature because some of its alleles—particularly 9.3—show marked frequency differences among populations, no single locus was interpreted here as a stand-alone ancestry marker. In the present context, loci such as TPOX and SE33 are better understood as contributors to the overall diversity profile of the Black Caribbean sample than as independent indicators of specific continental ancestry components [40,41,42].

### 4.2. Hardy–Weinberg Equilibrium and Population Structure

The absence of significant HWE deviations after Bonferroni correction supports the genetic homogeneity and panmictic structure of the sampled population. The single marginal deviation at D12S391 (*p* = 0.0320) is likely attributable to sampling variability rather than genuine disequilibrium, given that it did not survive correction and that the direction of deviation was consistent with random allele frequency sampling in a finite population. This overall picture of equilibrium is broadly consistent with prior forensic STR reports in Afro-descendant Caribbean populations from Honduras [43,44], although formal inference regarding founder effects would require denser marker systems than the present 23 STR panel.

We note that the Bonferroni correction applied here prioritizes specificity over sensitivity, minimizing false-positive identification of problematic loci at the cost of reduced statistical power to detect subtle deviations. For the primary purpose of this study, which is the construction of a reliable forensic reference database, this conservative stance is appropriate. Researchers employing this dataset for population structure inference, however, should be aware that mild sub-structure or weak HWE departures may escape detection with N = 100. Future studies incorporating larger sample sizes and genome-wide markers will provide greater resolution for demographic modelling.

### 4.3. Forensic Statistical Parameters

The combined forensic performance of the 23-locus panel in the Black Caribbean population is exceptional. The combined PD (>99.9999%) and combined CE (>99.9999%) confirm the panel’s suitability for both criminal identity investigations and kinship analyses in this population. Individual-locus PD values ranged from 0.8310 to 0.9806, ensuring that even the least polymorphic marker contributes meaningful discriminatory power to the multilocus product. Twenty-two of 23 loci surpassed PIC = 0.60, confirming that most markers in the panel are highly informative for forensic discrimination in this population.

These results underscore the importance of utilizing a population-appropriate database when computing forensic statistics for Black Caribbean individuals. Using a non-representative database such as the generic Hispanic allele frequencies from the FBI CODIS reference or the Honduran mestizo dataset, would systematically misrepresent allele frequencies at loci where divergence between populations exceeds 0.20 (e.g., TH01 allele 7, D2S441 allele 14, SE33 high-number alleles), potentially inflating or deflating the strength of forensic evidence and thereby jeopardizing the reliability and fairness of judicial proceedings [2,3,4].

A critical qualification must accompany any forensic use of a population-specific database. As Chambers et al. [19] argued in their critique of the NRC II recommendations, restricting the reference population to a narrowly defined ethnic group is statistically appropriate only when independent evidence establishes, with reasonable certainty, that the perpetrator belongs to that group. In general casework, the alternative-suspect population must represent all plausible contributors to the evidence, not a pre-selected cohort [3,19]. Furthermore, even within a historically cohesive community such as the Honduran Black Caribbean, some degree of admixture with neighbouring populations is expected; no database can therefore be assumed to represent a genetically closed unit. The Black Caribbean allele frequency database reported here is most appropriately used: (i) in paternity testing and kinship analyses where the ethnic affiliation of all parties is independently documented; (ii) as one component of a composite, demographically weighted reference database in regions where Black Caribbean individuals constitute a substantial fraction of the local population; and (iii) for within-group coancestry calibration (θ), as discussed in Section 4.4. Its application as the sole reference database in criminal casework should be restricted to cases where the ethnic composition of the alternative suspect pool is independently established and justified in writing in the case file [3,19].

### 4.4. Population Substructure and Theta Correction

The coancestry coefficient θ, closely related to FST, is used to adjust product-rule calculations for the non-independence of alleles in structured populations [3,35,45]. Honduran forensic laboratories currently apply a uniform θ = 0.01 across ethnic groups. Formal pairwise FST comparisons between the Black Caribbean population and other Honduran reference groups have not yet been performed because shared individual-level genotype data are not currently available. Nevertheless, published FST estimates for forensic STR markers [46], together with the observed differences in mean heterozygosity between the Black Caribbean and Honduran indigenous datasets, support consideration of a provisional and conservative theta framework. Accordingly, Table 2 should be interpreted as an exploratory reference for discussion and future validation, pending empirical pairwise FST estimation in Honduras.

Use of population-sensitive theta values may help keep statistical output conservative and better aligned with underlying population structure in forensic interpretation. However, the framework shown in Table 2 should be regarded as provisional until formal pairwise FST estimates become available for the Black Caribbean population relative to other Honduran ethnic groups. Any future operational adoption would require: (1) standardized documentation of ethnic affiliation in case files; (2) targeted training for forensic analysts on database selection and theta calibration; and (3) clear expert testimony guidelines for explaining theta corrections to judicial audiences. Empirical genome-wide and STR-based interpopulation comparisons will be necessary to refine this framework with greater precision.

### 4.5. Comparative Population Analysis

In this exploratory regional comparison, the Black Caribbean population showed the highest mean He (0.8150) and the highest mean allelic richness (10.48 alleles/locus) among the datasets considered. However, these cross-study comparisons are strictly descriptive and should not be interpreted as direct quantitative contrasts among populations, because the underlying studies were generated with only partially overlapping autosomal STR panels. In particular, the present study and the Honduran Tawahka and Lenca datasets used 23 loci, whereas the Guatemalan Ladino dataset was based on 15 STRs. Therefore, differences in diversity estimates may partly reflect marker-set composition in addition to population genetic history. Within these limitations, the Honduran Tawahka and Lenca populations exhibited lower diversity estimates, whereas the Guatemalan Ladino population occupied an intermediate position within this restricted comparative framework.

Despite the limitations imposed by non-identical marker panels, the observed pattern is broadly compatible with previously described continental gradients in STR diversity, in which sub-Saharan African populations tend to display broader allele spectra and higher heterozygosity than many non-African populations [47]. Comparison with West African reference populations [39] and Afro-descendant Caribbean datasets from Honduras [43] would further delineate the extent of genetic continuity or divergence attributable to island geography, admixture, and founder effects since the original forced migration.

Table 3 provides an exploratory regional comparison of mean heterozygosity, discriminatory power, and allelic richness across Central American populations typed with partially overlapping autosomal STR panels.

Several allele frequencies merit specific comment in the context of Central American forensic databases. At D22S1045, allele 16 which dominates the Tawahka (0.60) and Lenca (0.58) frequency spectra is present at a notably lower frequency in the Black Caribbean sample (0.2100), reflecting the different allele-frequency distributions typical of West African versus Amerindian populations. Similarly, at D2S441, allele 14 reaches its maximum frequency in this dataset (0.3350), exceeding values in all three Honduran groups studied to date, a pattern reported in several West African STR surveys [39]. These divergences reinforce the scientific and legal necessity of population-specific databases.

**Figure 2:**
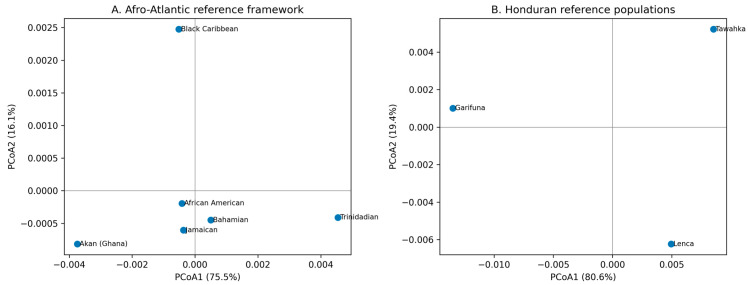
Two panel principal coordinates analysis (PCoA) based on pairwise FST distances (**A**) Afro-Atlantic comparative framework reconstructed from harmonized allele-frequency tables over 11 shared autosomal STR loci for the present Black Caribbean sample and the Akan (Ghana), African American, Bahamian, Jamaican, and Trinidadian reference populations. (**B**) Honduran comparative framework based on published pairwise FST values for the Lenca, Tawahka, and Garifuna populations typed with comparable 23-locus datasets. Together, the two panels place the Honduran Black Caribbean population within a broader Afro-Atlantic context while preserving methodological comparability within the Honduran reference set.

In Figure 2 at Panel A the Honduran Black Caribbean sample clustered within the broader Afro-Atlantic space and plotted closest to the African American, Bahamian, and Jamaican reference populations, while the Akan (Ghana) and Trinidadian samples occupied more peripheral positions. PCoA1 and PCoA2 explained 75.5% and 16.1% of the total inertia, respectively. In Panel B, the Honduran reference populations showed the expected internal arrangement, with Lenca and Tawahka positioned closer to each other than either was to Garifuna. In this ordination, PCoA1 explained 80.6% of the total inertia and PCoA2 explained 19.4%. Taken together, the two panels support interpretation of the Honduran Black Caribbean population within a broader Afro-Atlantic comparative framework while retaining the Honduran population structure context [6,7,19,36,44].

A two-panel approach from Figure 2 is methodologically preferable to a single pooled ordination because it avoids forcing a unified matrix from studies that do not all provide fully homologous allele-frequency tables across the same marker set, while still enabling direct visual integration of the Afro-Atlantic and Honduran comparative contexts.

### 4.6. Forensic and Broader Applications

Beyond its immediate forensic utility, this dataset has relevance for several applied and research contexts. In forensic casework, it provides a population-appropriate reference for random match probability (RMP) and paternity index (PI) calculations involving Black Caribbean individuals, subject to the condition that the ethnic composition of the alternative suspect pool justifies its use as a primary or contributing reference [3,19].

This population has been underrepresented in the current Honduran forensic framework, and the availability of population-specific data extends the statistical options for practitioners, particularly in paternity testing where the ethnic affiliation of all parties can be independently confirmed. The availability of population-specific allele frequencies is expected to improve the precision of likelihood calculations, particularly at loci showing substantial frequency differences relative to existing Honduran reference datasets. From a population-genetic perspective, the dataset also contributes a useful baseline for future studies of diversity, demographic history, and interpopulation differentiation within Afro-descendant and other ethnically differentiated populations of Honduras and the wider Central American region.

### 4.7. Limitations and Considerations

Several limitations constrain the scope of the present findings. First, a sample size of N = 100, while consistent with published forensic population studies [6,7,16,48], limits precision in estimating the frequencies of rare alleles and may underpower formal tests of mild population substructure. Second, sampling was restricted to the department of Islas de la Bahía and does not capture Black Caribbean communities on the mainland northern coast, where some degree of admixture with mestizo and Garífuna populations may have altered allele frequencies. Third, the absence of genome-wide genotyping data precludes formal admixture modelling; the degree of European or Amerindian ancestry components in this sample remains to be quantified. Fourth, formal pairwise FST calculations with other Honduran populations await shared individual-level genotype access from prior studies. Future work incorporating a geographically expanded sampling frame, larger cohort sizes, and SNP-based ancestry inference will address these limitations and further delineate the population structure of this historically important community.

### 4.8. Implementation and Future Directions

The Black Caribbean allele frequency database presented here will be incorporated into the operational workflow of the Forensic Medicine Directorate of Honduras’s Public Ministry. A formal implementation protocol specifying database selection criteria based on self-reported or genealogically established ethnic affiliation will be developed in parallel with training materials for forensic analysts covering appropriate database selection, theta calibration, and expert testimony best practices.

This study constitutes part of a broader programme (2025–2028) jointly coordinated by the UNAH Faculty of Medical Sciences and the Public Ministry of Honduras to characterize all nine Honduran indigenous and Afro-descendant peoples using the PowerPlex Fusion 6C System. The Garífuna, Miskito, Pech, Tolupán, and Chortí populations remain priority targets for forthcoming studies, with target sample sizes of N = 100 per group. An expanded mestizo dataset (N = 200) spanning both northern and central regions is likewise planned. Collectively, these initiatives aim to achieve comprehensive STR database coverage for all major Honduran ethnic communities by 2028, establishing a regionally unmatched framework for equitable forensic genetic services.

## 5. Conclusions

This investigation delivers the first population-specific genetic characterization of the Honduran Black Caribbean (Negro Inglés) community, documenting allele frequencies for 23 autosomal STR loci in 100 unrelated individuals. The panel revealed high within-population genetic diversity, mean He = 0.8150, mean alleles per locus = 10.48; consistent with the West African demographic heritage of this community and substantially exceeding the diversity documented in Honduran indigenous groups. No locus exhibited a significant departure from Hardy–Weinberg equilibrium after Bonferroni correction, confirming the statistical adequacy of the dataset for forensic applications. The combined PD and combined CE both surpassed 99.9999%, underscoring the exceptional discriminatory and exclusionary power of the 23-locus PowerPlex Fusion 6C panel in this population.

The dataset fills a critical gap in Honduras’s forensic genetic infrastructure, providing the analytical foundation for population-appropriate likelihood-ratio calculations in criminal casework and paternity testing involving Black Caribbean individuals. Beyond forensic applications, the data contributes to the characterization of Afro-descendant genetic diversity in Central America and serves as a baseline resource for population-genetic, anthropological, and medical genetics research. Aggregated allele frequencies are released as Supplementary File S1 to promote methodological transparency and facilitate comparative population analyses, whereas access to de-identified individual genotype records is subject to institutional authorization. This study exemplifies the scientific and ethical commitment to ensuring that every Honduran community, irrespective of ethnic background has access to accurate and culturally sensitive forensic genetic services.

## Figures and Tables

**Table 1:** Per-locus genetic diversity and forensic statistics for 23 autosomal STR loci in the Black Caribbean population of Honduras (N = 100). Na: number of observed alleles; He: expected heterozygosity; Ho: observed heterozygosity; PD: power of discrimination; CE: chance of exclusion; PIC: polymorphic information content; *p* (HWE): exact-test *p*-value for Hardy–Weinberg equilibrium. Asterisk (*) denotes nominal significance (*p* < 0.05) not retained after Bonferroni correction (α = 0.0022).

Locus	Na	He	Ho	PD	CE	PIC	*p* (HWE)
D3S1358	10	0.7706	0.7800	0.9072	0.5625	0.7306	0.1015
D1S1656	13	0.8447	0.8400	0.9536	0.6753	0.8228	0.3730
D2S441	9	0.7725	0.8000	0.8992	0.5990	0.7337	0.4915
D10S1248	6	0.7541	0.7600	0.8956	0.5270	0.7163	0.2045
D13S317	7	0.6541	0.6300	0.8310	0.3284	0.5999	0.9030
Penta E	15	0.9042	0.9100	0.9746	0.8159	0.8912	0.7160
D16S539	7	0.7960	0.8200	0.9210	0.6367	0.7601	0.9835
D18S51	12	0.8732	0.8700	0.9616	0.7346	0.8549	0.7090
D2S1338	12	0.8858	0.8200	0.9668	0.6367	0.8696	0.0945
CSF1PO	8	0.7922	0.7900	0.9192	0.5806	0.7569	0.8325
Penta D	11	0.8843	0.8600	0.9683	0.7147	0.8678	0.9155
TH01	6	0.7321	0.7800	0.8774	0.5625	0.6889	0.1015
vWA	9	0.8139	0.7900	0.9326	0.5806	0.7829	0.7420
D21S11	13	0.8561	0.8300	0.9541	0.6559	0.8348	0.5200
D7S820	8	0.8069	0.8300	0.9254	0.6559	0.7738	0.3810
D5S818	10	0.6946	0.6600	0.8568	0.3691	0.6423	0.3610
TPOX	7	0.7823	0.7900	0.8972	0.5806	0.7436	0.1200
D8S1179	8	0.7998	0.7700	0.9270	0.5446	0.7697	0.2485
D12S391	10	0.8390	0.8000	0.9427	0.5990	0.8144	0.0320 *
D19S433	13	0.8709	0.8600	0.9616	0.7147	0.8539	0.1310
SE33	24	0.9350	0.9100	0.9806	0.8159	0.9261	0.3850
D22S1045	9	0.8157	0.8100	0.9311	0.6177	0.7851	0.6885
FGA	14	0.8673	0.8400	0.9623	0.6753	0.8482	0.1530
Mean ± SD	10.48 ± 3.85	0.8150 ± 0.0664	0.8065 ± 0.0637	0.9281 ± 0.0382	0.6167 ± 0.1125	0.7855 ± 0.0770	—

**Table 2:** Provisional and conservative coancestry coefficient (θ) framework for forensic calculations in Honduras involving the Black Caribbean reference database.

Scenario	Recommended θ	Justification
Within Black Caribbean (suspect and reference both confirmed Black Caribbean)	θ = 0.01	Conservative; within-population FST estimated ≤0.005; accounts for potential cryptic relatedness
Within Honduran indigenous groups (Tawahka, Lenca)	θ = 0.01	Established standard for these populations
Within the Honduran mestizo	θ = 0.01	Existing standard; appropriate for admixed, urbanized population
Black Caribbean vs. mestizo or indigenous (unknown ethnicity)	θ = 0.03	Reflects substantial between-group FST; conservative for cross-population comparisons
Black Caribbean vs. Asian/European populations (international cases)	θ = 0.05–0.10	Reflects large inter-continental genetic distances
Unknown ethnicity (no information available)	θ = 0.03	Intermediate conservative value balancing statistical utility and protection against substructure bias

**Table 3:** Exploratory comparison of forensic diversity parameters across Central American populations genotyped with partially overlapping autosomal STR panels.

Population	N	Mean He	Mean PD	Mean Na/Locus	Reference
Black Caribbean (Honduras)	100	0.8150	0.9281	10.48	Present study
Tawahka (Honduras)	100	0.7385	0.8771	8.61	Zuniga et al. [7]
Lenca (Honduras)	100	0.7425	0.8815	8.91	Zuniga et al. [6]
Maya (Guatemala)	155	0.7104	0.8534	7.83	Cardoso et al. [48]
Ladino (Guatemala)	130	0.7656	0.8945	9.45	Aguilar-Velázquez et al. [16]

Note: Cross-population comparisons are strictly exploratory because the reference studies were not based on identical STR panels. The present study, as well as the Honduran Lenca and Tawahka datasets, used 23 autosomal STR loci, whereas the Guatemalan Maya dataset was based on 16 autosomal STRs and the Ladino dataset on 15 STRs. Therefore, differences in mean He, mean PD, and mean Na/locus should not be interpreted as directly equivalent estimates across all populations.

## Data Availability

Supplementary File S1 (aggregated allele frequencies) is provided as open Supplementary Material. The actual de-identified individual genotype records from the 100 enrolled participants are held under joint custodianship of the Forensic Medicine Directorate, Public Ministry of Honduras. Requests for access should be directed to the corresponding author and must receive written authorization from both entities.

## Supplementary Materials

The following supporting information can be downloaded at: https://www.mdpi.com/article/10.3390/genes17050496/s1, Supplementary File S1—Population genetic data for 23 autosomal STR loci (PowerPlex Fusion 6C System) in the Black Caribbean (Negro Inglés) population of Honduras (Excel format). This file contains per-locus allele designations, frequencies, and summary statistics (N, Ho, He, PD, CE, PIC, MAF, HWE *p*-value).

## Abbreviations

The following abbreviations are used in this manuscript:

AMOVA	Analysis of Molecular Variance
CE	Chance of Exclusion
CEIB/UIC	Biomedical Research Ethics Committee/Research Unit (committee acronym as cited in the manuscript)
CI	Confidence Interval
CODIS	Combined DNA Index System
DNA	Deoxyribonucleic Acid
ESS	European Standard Set
FBI	Federal Bureau of Investigation
FCM	Faculty of Medical Sciences
FST	Fixation Index
FTA	Flinders Technology Associates
GITAD	Ibero-American Working Group on DNA Analysis
He	Expected Heterozygosity
Ho	Observed Heterozygosity
HWE	Hardy–Weinberg Equilibrium
IBS	Identity by State
ICIMEDES	Institute for Research in Medical Sciences and Right to Health
IRB	Institutional Review Board
ISFG	International Society for Forensic Genetics
MAF	Minimum Allele Frequency
N	Sample Size
Na	Number of Observed Alleles
PCoA	Principal Components Analysis
PCR	Polymerase Chain Reaction
PD	Power of Discrimination
PI	Paternity Index
PIC	Polymorphic Information Content
RMP	Random Match Probability
SD	Standard Deviation
STR	Short Tandem Repeat
UNAH	National Autonomous University of Honduras
